# Antisepsis in Veterinary Practice1Read before the Missouri Valley Veterinary Association, June, 1899.

**Published:** 1899-08

**Authors:** H. G. Patterson

**Affiliations:** St. Joseph, Mo.


### THE PRACTICABILITY OF ANTISEPSIS IN VETERINARY PRACTICE.[1]

By H. G. Patterson, D.V.S.,
ST. JOSEPH, MO.

This subject may be considered in relation to the favorable and unfavorable conditions peculiar to the field of veterinary surgery. At a casual glance the subject seems to present the former in a very insignificant proportion indeed, and, by the

[1] Read before the Missouri Valley Veterinary Association, June, 1899.

most careful review of the question, we must admit that the latter constitute a most formidable array.

The fact that our patients are not amenable to reason constitutes one serious obstacle to the ready application of the technique of asepsis even in our simpler operations. Could we place our subject in proper position by a gentlemanly request to "Sit down here, now;" or "Just lie down here on this table;" or "Turn this way, or that way;" or could we induce him to take the anæsthetic by a kindly suggestion as to quiet, regular breathing, closed eyes, etc., it would place us one long stride in advance of our present situation. It is true that gentleness and patience, with a certain firmness and confidence in our superiority of mind over that of any lower animal, go far toward controlling the most nervous or vicious; but the instant we inflict pain we arouse the instinct of self-preservation and, inseparable from it, the effort to escape, or the most vigorous defensive measures the victim is capable of, and restraint by force is our only resource. While restraint is unpleasant, unsatisfactory, even when applied in the most scientific and skilful manner, it is not entirely devoid of danger to the patient and to those who must handle him.

The difficulty in retaining suitable protective dressings confronts us immediately on concluding an operation. The external conformation and the hairy covering render the application of bandages or adhesive strips a trying task, and, if not displaced by the struggles incident to the animal's release from the mechanism by which it has been controlled, dressings are at once regarded as an infringement on personal liberty not to be tolerated, and the ingenuity in undoing our finest handiwork is only equalled by the opportunities afforded by the ignorance and stupidity of the average attendant in whose care our dumb friend must be left. And right at this point a most trying and difficult position confronts us. The majority of us are compelled to leave our patients to the tender mercies of a class of men who, by their environment and social conditions, are, as a rule, neither intellectually nor morally desirable as nurses. Contend as we may for the equality of American citizens, the fact remains that a man who works long hours, frequently exposed to cold and storms, held responsible for accidents whether due to his carelessness or not, and, with all, overworked and underpaid, develops habits inimical to the service required and necessary for the proper care of veterinary patients. It is also impossible to impress such men with the importance of absolute cleanliness in their service. Filthy personal habits, if nothing else, render them unteachable in this line. If fortunate enough to escape all these pitfalls we still have the unsanitary surroundings, both in the city and country practice. Filthy stalls, urine-soaked, foul-smelling, without ventilation, or, if ventilated, at the expense of warmth and protection from storms.

These are stern facts, not dreams; we see them every day, and yet I am convinced that aseptic surgery is not only practicable, but, in the interests of our patients, our patrons, our pocket-book, and our profession, profitable and indispensable. To offset in some measure this deplorable list of adverse conditions, we have, on the other hand, the simplicity of living, absence of vicious habits, rarity of nervous complications, and, above all, an inherent power of resistance to infective germs. Consider the almost absolute immunity of the horse to such diseases as anthrax, tuberculosis, erysipelas, etc. Give a surgeon such conditions in his treatment of the human family, and surgery is robbed of half its dangers. Consider, again, the number of ovariotomies annually perpetrated (I believe this is the proper word, considering the skill of the parties usually employed in such work) on heifers and other animals, with no attempt at even the ordinary cleanliness observed by butchers, and with a very small per cent. of fatalities, and we have infallible proof of this resistance to pathogenic germs. How much more may we not accomplish by the strict adherence to the principles of asepsis and antisepsis? Discard the filthy stable-bucket, the dirty water, and greasy sponge; sterilize our instruments, sponges, hands, and prepare thoroughly our field of operation; in short, observe the full technique as approved by out best surgeons, and, notwithstanding so much that seems to threaten failure, results will be both surprising and gratifying.

I have refrained from any discussion of the comparative value of antiseptics. The careful and thorough application of the principles to the case in hand is of much more importance. If we cleanse everything by heat, and apply good soap, brush, razor, and elbow-grease, we are very near the goal. The selection of the antiseptic is and always will remain much a matter of choice and custom, or individual experience. Whenever the process of bacterial infection exists, whether it be in external wounds or in internal viscera, the use and administration of antiseptics is highly indicated, and, if directed timely and with proper judgment and selection, nothing but decided benefit can accrue from their use.

I would deem it needless excess to indulge in a recitation of the application of antiseptics to external wounds and trauma before this intelligent audience, but permit me to call your attention to a field of application which I believe is not recognized by very many veterinarians. I refer generally to the internal antiseptics, but particularly to their use in the treatment of intestinal disorders, especially those characterized by the presence of undue quantities of gas. Here antiseptics work marvellously favorable changes, and the administration of one good antiseptic will do more good than all the sixteen or thirty-two-ounce mixtures from the use of which animals are being killed every day. Numerous good antiseptics might be used, but my favorite in such conditions is carbolic acid in doses of ten to twenty drops given with water and glycerin every half or one hour, according to the urgency of the case. Salicylic acid in drachm doses is also an excellent antiseptic in such cases, but in my opinion not equal to carbolic acid. The sterilization of the urinary passages by the internal administration of antiseptics is also a reputable procedure. Whenever septic disease not due to foreign bodies or concentration of this set of organs exists, boric acid in drachm doses exhibited four to five times daily, together with the sedative action of fluid extract of buchu, is the treatment *par excellence.*

I will not take up any more of your time in speaking of the special administration of antiseptics, for to remember their general applicability is but to know their use in special affections.

*Discussion.*

Dr. Stewart: Antiseptic surgery is a subject in which I am very much interested, and one which the paper has quite happily introduced The surgeon who does not employ antiseptics in his practice is certainly laboring at a great disadvantage, and the range of its application should constantly increase. In many surgical and accidental wounds where it did not seem advisable to close the wound entirely, it was my practice to employ an old-time agent sometimes called white lotion. This compound was made many years before antisepsis was thought of, yet its antiseptic action, as well as its curative action, is well established. With reasonable care and cleanliness the veterinary surgeon can secure highly satisfactory results in the treatment of nearly all surgical wounds, and it seems strange to me that so few surgeons make full use of antiseptics in their everyday practice. There is one operation to which I will allude where the results for me have been most gratifying, healing by first intention being commonly secured. I refer to the ordinary neurectomy for navicular and other pedal lamenesses. I would like to hear what Dr. Hansen has to say on the subject.

DR. HANSEN: I did not come here to talk, but must say that Dr. Patterson's point as to the power of resistance in the horse to the germs of disease is very patent. My experience in veterinary surgery certainly upholds his paper.

